# Localization of hRad9 in breast cancer

**DOI:** 10.1186/1471-2407-8-196

**Published:** 2008-07-11

**Authors:** Vivian Chan, US Khoo, MS Wong, Ken Lau, Dacita Suen, George Li, Ava Kwong, TK Chan

**Affiliations:** 1 University Department of Medicine, Queen Mary Hospital, Hong Kong, China; 2 Department of Pathology, The University of Hong Kong, Queen Mary Hospital, Hong Kong, China; 3 Department of Surgery, Queen Mary Hospital, Hong Kong, China

## Abstract

**Background:**

*hRad9 *is a cell cycle checkpoint gene that is up-regulated in breast cancer. We have previously shown that the mRNA up-regulation correlated with tumor size and local recurrence. Immunohistochemical studies were made to better define the role of *hRad9 *in breast carcinogenesis.

**Methods:**

Localisation of hRad9 protein were performed on paired tumor and normal breast tissues. Immunoblotting with and without dephosphorylation was used to define the protein isolated from breast cancer cells.

**Results:**

Increased hRad9 protein was observed in breast cancer cells nucleus compared to non-tumor epithelium. This nuclear protein existed in hyperphosphorylated forms which may be those of the hRad9-hRad1-hHus1 complex.

**Conclusion:**

Finding of hyperphosphorylated forms of hRad9 in the nucleus of cancer cells is in keeping with its function in ameliorating DNA instability, whereby it inadvertently assists tumor growth.

## Background

Human Rad 9 (*hRad9*) was originally identified as a structural homologue of yeast schizosaccharomyces pombe *rad 9*, which can partially rescue the sensitivity of *rad 9 *null yeast to hydroxyurea, radiation damage and the associated checkpoint defects [1]. One would expect that this gene functions as a tumor suppressor gene. This is supported by the finding that *hRad9 *is a negative co-regulator, suppressing androgen receptor activation in prostate cancer cells [2] and that the mammalian prologue *hRad9B *is expressed predominantly in the testis and at a reduced level in testicular tumors compared to normal adult testis [3].

Normally, structural damage of DNA by endogenous and environmental agents is followed by replication checkpoint arrest at the G2/M transition in order to allow for repair before proceeding in the cell cycle. Also with excessive DNA damage, apoptosis of the cell occurs [4]. The loss of proper response to DNA damage leads to genomic instability such as gene mutations, incomplete replication and the loss or gain of chromosomes to future generations. These genetic aberrations may cause loss of growth inhibition in normally quiescent cells and result in carcinogenesis [5].

However, in established cancer cells, such DNA repair system may prevent further DNA damage in their progeny and decrease in apoptosis resulting in enhancement of tumor growth. Hence, the phosphorylated forms of *hRad9 *have been identified in the tumor cells nucleus of non-small cell lung carcinoma (NSCLC) and increased expression of *hRad9 *was related to increased tumor cell proliferation [6]. However, sequencing of the *hRad9 *gene failed to identify any mutation although a non-synonymous single nucleotide polymorphism (SNP), His239Arg, was found to be associated with lung adenocarcinoma [7]. We have also demonstrated previously overexpression of *Rad9 *mRNA in a number of primary breast tumors and the increased *Rad9 *mRNA was correlated with an increased risk of local recurrence and tumor proliferation, suggesting that *Rad9 *is an oncogene in breast cancer [8].

hRad9 is a nuclear protein that interacts with hRad1 and hHus1 to form a hetero-trimeric complex (the 9-1-1 complex) which is then loaded onto DNA [9]. The C-terminal domain of hRad9 contains phosphorylation sites and hyperphosphorylation of hRad9 occurs in response to DNA damage [10]. It also contains a nuclear localization sequence (NLS) that targets the hRad9 protein into the nucleus [11]. Presumably the C-terminal domain is essential for transport of the 9-1-1 complex from the cytoplasm into the nucleus, for activation of the G2 checkpoint signalling cascade [12]. Additionally, hRad9 contains a Bcl-2 homolog 3 (BH3)-like domain at its NH_2 _terminus that can bind the anti-apoptotic proteins Bcl2 and Bcl-xL, thereby promoting apoptosis when DNA repair fails [13]. Thus, in addition to its checkpoint control function, *hRad9 *may play a role in regulating apoptosis.

To further examine the role of hRad9 in breast cancer cells, we now report on the histologic expression of the hRad9 protein and its different molecular forms in primary breast cancer and normal tissues.

## Methods

Thirty-seven sets of breast tumor and adjacent normal breast tissues obtained during surgical resection were used in this study; with ethics approval from the Institutional Review Board of the University of Hong Kong/Hospital Authority Hong Kong West Cluster (Ref No. UW 06-036 T/1061) and according to the Declaration of Helsinki. Upon surgical resection, part of the samples was immersed in RNAlater (Ambion, Inc., Austin, Texas) and incubated overnight at 4°C before long-term storage at -80°C. Other portions of these samples were fixed with 10% (volume/volume) neutral-buffered formalin overnight and then embedded in paraffin.

Of the 37 paired breast tumor and adjacent normal breast tissues, 22 cases were previously determined by quantitative PCR [8] as having a relative *hRad9 *mRNA level of > 2 in the tumor sample (compared to adjacent normal breast tissue) and 15 cases having *hRad9 *mRNA level < 2. These were designated as Group 1 and Group 2 respectively.

### Immunohistochemistry

Immunohistochemical studies were performed on paraffin sections using the avidin-biotinylated peroxidase complex method (Dako Cytomation A/S, Glostrup, Denmark) according to the manufacturer's instruction. Briefly, sections were deparaffinised and rehydrated through ethanol series (99%, 95% and 80% Ethanol). Antigen retrieval was made by heating for 1 min in Tris/EDTA buffer in a pressure cooker. Sections were incubated for 20 min with 3% hydrogen peroxide in water, to remove endogenous peroxidase activities and for a similar period with diluted blocking solution (10 mg bovine serum albumin, 10% normal goat serum and 1 μl Triton X-100 in Tris buffer saline, TBS). The sections were initially incubated with diluted (1:75) Rad9 antibody (M-389, Santa Cruz Biotechnology, CA, USA) at 4°C overnight, and rinsed with TBS, before incubation with a biotinylated second antibody solution (KO492, Dako, 1:100 dilution) at 37°C for 30 min. The bound antigen was visualized using streptavidin-biotin-hydrogen peroxide complex with 3, 3'-diamobenzidine tetrachloride (DAB) as substrate and counterstained with Mayer's haematoxylin. Staining distribution and the intensity of staining were scored separately by two persons, blinded to the pathology of the sections. Each slide was scored for cytoplasmic and nuclear staining of the cancer and normal cells. Semiquantitative grading was made according to the following scores: 0 (negative); 1+ (weak positive), 2+ (moderate positive) and 3+ (strong positive).

### Western immunoblotting

One hundred mg of frozen tissue was homogenised in 500 μl of Laemmli sample buffer (Biorad, CA, USA) containing 100 mM dithiothreitol at 4°C using a Polytron generator. Extracts were centrifuged at 10,000 g × 5 min at 4°C to remove debris and 10 μl of the supernatant heated in a boiling water bath for 5 min before subjecting to sodium dodecyl sulphate-polyacrylamide gel electrophoresis (0.1% SDS-10% PAG). The proteins were transferred to nitrocellulose membrane by electroblotting. The membrane was blocked with 5% non-fat milk, 0.05% Tween 20 in phosphate buffer saline for 1 hr at room temperature, to reduce non-specific protein binding. It was then incubated with anti-Rad9 (at 1:1000 dilution, Santa Cruz Biotechnology) in a sealed bag at 4°C overnight, followed by incubation with a second antibody (1:3000 dilution), again at 4°C for 1 hr before visualization using the ECL system (GE Healthcare Ltd, Little Chalfont, UK).

### Phosphatase experiment

To demonstrate that hRad9 protein in breast cancer cells is in phosphorylated forms, 100 mg amounts of frozen tissue were extracted as previous in the presence of protease inhibitors (protease inhibitor cocktail, set III; EMD Biosciences Inc, CA, USA). Forty mg of protein were treated with λ phosphatase (New England Biolabs, Beverly, MA, USA) in accordance with the manufacturer's instruction. The treated samples were heated at 95°C × 5 min, then on ice × 5 min before subjecting to SDS-10% PAG electrophoresis. Electroblotting and immunostaining were performed as described previously (vide supra).

## Results

### Localization of hRad9 protein in breast samples

**Figure 1:**
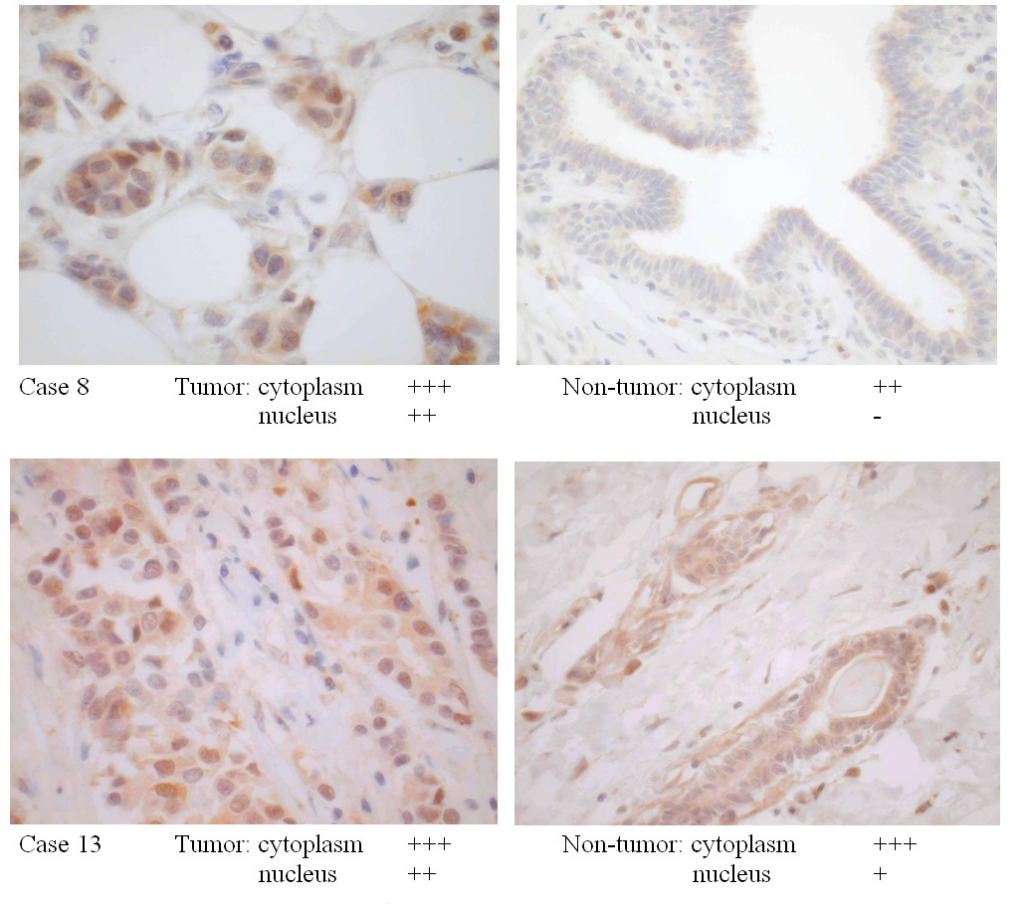
**Immunohistochemical stain for hRad9 protein in two breast samples **(case 8 and case 13) taken during surgical resection. Paired tumor (left panel) and normal (right panel) breast tissue was studied. In both cases, tumor cell nucleus stained more heavily (2+) compared to normal cell nucleus (0 and 1+).

Figure 1 showed tumor cells and non-tumor cells stained for hRad9 protein.

Whilst hRad9 protein was observed in similar amounts in the cytoplasm of both normal cells and cancer cells in all specimens studied, the staining in the nucleus of cancer cells were significantly higher (p < 0.001) and the combined cytoplasmic and nuclear scores were also higher in the cancer cells (p = 0.002) (Table 1). Furthermore, the nuclear score found in tumor samples with increased *hRad9 *mRNA (Group 1), was significantly higher compared to tumor samples with normal *hRad9 *mRNA level (Group 2), while the cytoplasm scores were similar (Table 2).

**Table 1:** Semiquantitative scores of immunoreactive hRad9 protein in sections of all tumor samples studied

	N	Cytoplasmic score	Nuclear score	Cytoplasmic + nuclear score
Normal cells	37	1.402 ± 0.532*	0.111 ± 0.340	1.513 ± 0.741
Cancer cells	37	1.333 ± 0.560	0.861 ± 0.661	2.208 ± 1.091
Paired t test		0.502	7.314	3.382
p		0.0619	0.000	0.002

*Mean ± SD

**Table 2:** Immunoreactive hRad9 protein scores in two groups of breast cancers

	N	mRNA ratio* (Cancer/normal cells)	Cytoplasmic score	Nuclear score	Cytoplasmic + nuclear score
Group 1	22	5.375 ± 2.443**	1.48 ± 0.663	1.16 ± 0.497	2.659 ± 1.039
Group 2	15	1.383 ± 0.435	1.20 ± 0.414	0.47 ± 0.667	1.666 ± 0.975
t		7.490	0.436	3.619	2.921
p		< 0.001	0.16	0.001	0.006

*Quantitative PCR for these samples was performed in a previous study [8].

**Mean ± SD.

### Western blot and λ phosphatase treatment

**Figure 2:**
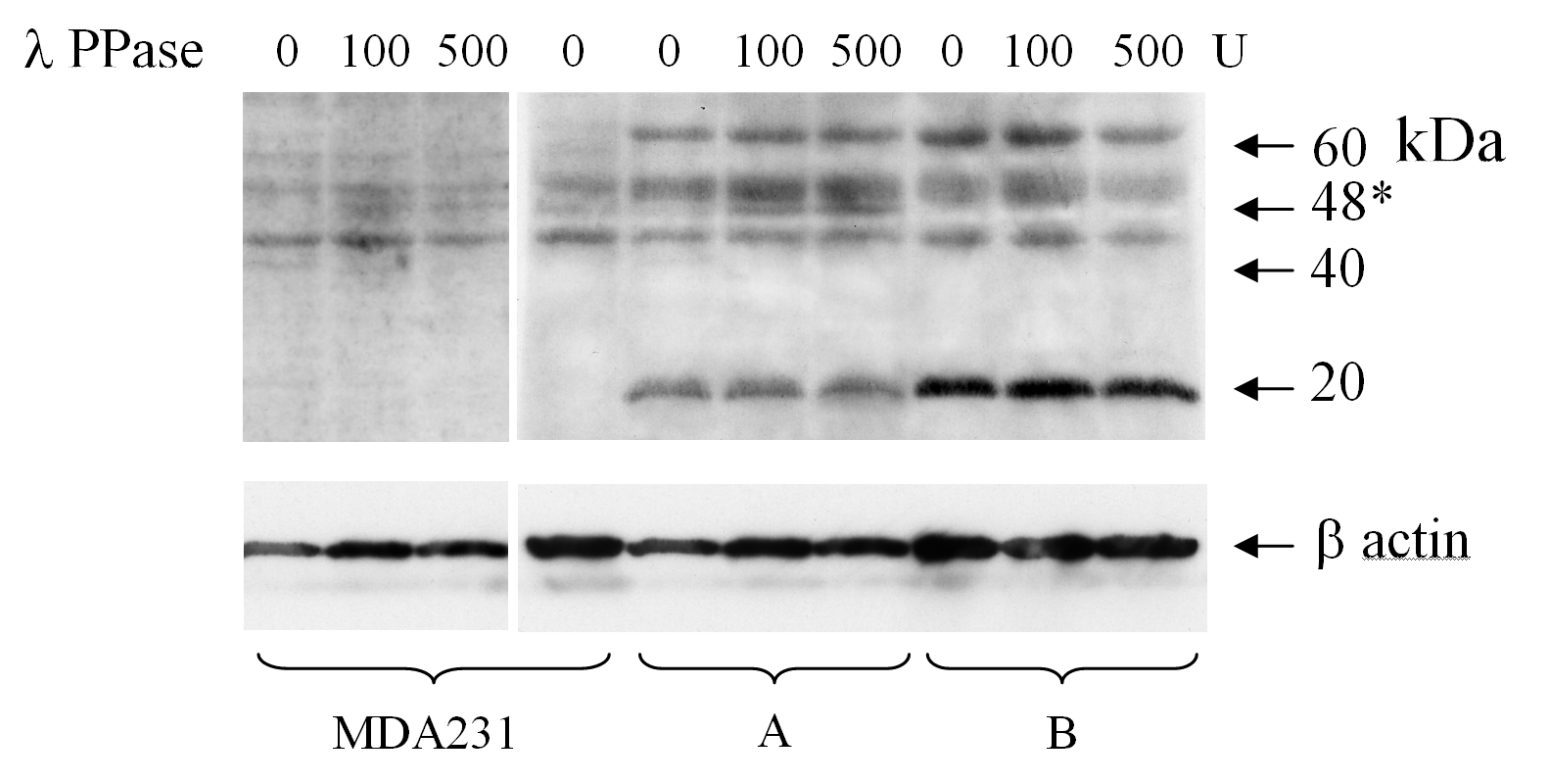
**Western blot showing hRad9 protein **extracted from MDA231 breast cancer cells, breast tumor sample with nuclear staining of hRad9 (A) and a control tumor sample with no nuclear staining (B). The relative position for molecular weights (in KDa) are indicated on the right. Protein extracts were treated with λ phosphatase (100 and 500 units respectively).

Western blot analysis showed at least four different species of hRad9 protein in a breast tumor sample with positive nuclear staining, with molecular weight ranging from 65 to 45 KDa. Whereas in MDA-231 breast cancer cell line, the hRad9 protein was mainly 50 and 45 KDa. Dephosphorylation of hRad9 protein extracted from both the tumor sample with nuclear staining and the MDA-231 cancer cell line with λ phosphatase yielded an additional 48 KDa species. The control tumor sample (which had no nuclear hRad9 staining), did not produce any 48 KDa species even upon treatment with 500 units of λ phosphatase (Figure 2).

## Discussion

Breast cancer cells were demonstrated in this study to have significantly increased hRad9 protein staining in their nuclei. This is especially marked in those shown to have increased *hRad9 *mRNA levels. We have shown previously that, clinico-pathologically, increased tumor size and chance of local recurrence were positively related to mRNA level of the tumor [8]. Hence the higher levels of hRad9 protein in the tumor nuclei may indicate a poorer clinical outcome of the breast cancer. This finding is similar to Maniwa et al.'s work on NSCLC [6]. Concomitant with the increased nuclear staining of hRad9, Maniwa's group demonstrated an increase in phosphorylated checkpoint kinase-1 (ChK1), suggesting that this is a physiologic response of the DNA damage checkpoint signaling pathway to genetic aberrations that occur in tumor cells. Maniwa et al. initially postulated that a mutated form of hRad9 may be present in the malignant cells, similar to the presence of mutated p53 that were over-expressed in the nucleus of primary lung cancer [14]. However, sequencing of *hRad9 *gene in NSCLC samples did not reveal any mutation [7]. We have previously shown that increased in *hRad9 *messenger in breast cancer was either due to gene amplification or hypermethylation in intron 2 of *hRad9 *gene, a silencer [8], resulting in increased transcription. Thus it would appear that the increased expression of *hRad9 *in tumor cells nuclei in both lung and breast cancers may serve to enhance DNA repair.

In human breast cancer cells, hRad9 exists in various forms, with molecular weights of 65, 50 and 45 KDa. In contrast, MDA-231 human breast cancer cell line contains the 50 and 45 KDa bands only. The theoretical molecular weight of hRad9 is 45 KDa [15] and the 65 and 50 KDa forms most likely represent hyperphosphorylated hRad9 and its hRad9-hRad1-hHus1 (9-1-1) complex. Upon dephosphorylation of this protein with λ phosphatase, an increasing amount of 50 KDa and a new 48 KDa form were noted. The 48 KDa form was only observed in MDA-231 cell line and in the breast tumor sample with nuclei staining (Figure 2; Panel A) and was absent in the tumor sample without nuclei staining (Figure 2; Panel B). Hence the larger hRad9 proteins, representing 9-1-1 complexes, are probably hyperphosphorylated as a cellular response to DNA damage in the nucleus [6]. Our findings of increased nuclear localization of hRad9 in breast cancer compared with non-tumor epithelium, which correlated with increased *hRad9 *mRNA expression, suggests that nuclear localization of hRad9 may be a response to DNA damage. It is most possible therefore that hRad9 protein functions in the nucleus of cancer cells to ameliorate DNA instability and inadvertently assists tumor growth. Further functional study of the *hRad9 *and its signalling pathway would be worthwhile to elucidate its role in breast cancer development and progression.

## Conclusion

Finding of hyperphosphorylated forms of hRad9 in the nucleus of cancer cells is in keeping with its function in ameliorating DNA instability, whereby it inadvertently assists tumor growth.

## Competing interests

The authors declare that they have no competing interests.

## Authors' contributions

VC devised the study, analysed data and prepared the manuscript. USK supervised the experimental work and helped in manuscript preparation. MSW and KL conducted the experiments. DS, GL and AK performed surgery and collected specimen. TKC analysed data and prepared manuscript.

## Pre-publication history

The pre-publication history for this paper can be accessed here:


